# Socioeconomic inequality as a predictor of unmet health needs in the older adult population of Serbia

**DOI:** 10.3389/fpubh.2024.1373877

**Published:** 2024-07-18

**Authors:** Nikola Savić, Svetlana Radević, Verica Jovanović, Nevena Ranković, Igor Lukić, Slobodanka Bogdanović Vasić, Branimirka Arandjelović, Biljana Bajić, Andrea Mirković, Aleksandra Arnaut, Borko Bajić, Svetlana Vukosavljević, Sanja Kocić

**Affiliations:** ^1^Faculty of Medical Sciences, University of Kragujevac, Kragujevac, Serbia; ^2^Faculty of Medical Sciences, Department of Social Medicine, University of Kragujevac, Serbia, Kragujevac, Serbia; ^3^Institute for Public Health of Serbia “Dr. Milan Jovanović Batut”, Belgrade, Serbia; ^4^Tilburg School of Humanities and Digital Sciences, Department of Cognitive Science, Tilburg University, Tilburg, Netherlands; ^5^Department of Medical Sciences, Academy of Vocational Studies Šabac, Šabac, Serbia; ^6^Faculty of Medicine, Department of Health Care, University of Novi Sad, Novi Sad, Serbia; ^7^Department of Health Promotion, Institute of Public Health of Montenegro, Podgorica, Montenegro; ^8^Faculty of Medical Sciences, Department of Dentistry, University of Kragujevac, Kragujevac, Serbia; ^9^Department for Health Ecology, Institute of Public Health of Montenegro, Podgorica, Montenegro

**Keywords:** unmet health needs, socioeconomic inequalities, older adult population, National Health Survey, Serbia

## Abstract

**Objectives:**

The aim of this paper is to assess the relationship between demographic and socioeconomic predictors and the unmet health needs of the older adult population in Serbia.

**Materials and methods:**

The study is part of the Population Health Survey of Serbia, which was conducted in the period from October to December 2019 by the Institute for Public Health of Serbia “Dr. Milan Jovanović Batut” and the Ministry of Health of the Republic of Serbia. The research was conducted on a representative sample of Serbian residents in the form of a cross-sectional study. For the purposes of this research study, data on senior citizens, aged 65 and older, were used.

**Results:**

Multivariate regression analysis of demographic characteristics that showed statistical significance in the univariate model as a whole explains between 4.2% (Cox & Snell R Square) and 5.9% (Nagelkerke R Square) of the variance of unmet health needs and correctly classifies 66.3% cases. Statistically significant demographic predictors were the region where the respondents live, level of education, and material condition. The results of the research show that the most dominant predictors of the unmet health needs of the older adult population are related to socioeconomic inequalities, financial reasons, and predictors related to the inaccessibility of health care.

**Conclusion:**

The results suggest that individual socioeconomic predictors have a great influence on the emergence of unmet health needs of the older adult population in Serbia. Every third older adult resident did not receive the necessary health care, most often due to financial constraints.

## 1 Introduction

Predictors influencing unmet health needs in the older adult population are numerous; these factors impact all determinants of health and the quality of life for this population in various ways (1). According to the World Health Organization’s definition, health needs can be scientifically identified deviations from health that require the application of preventive, curative, control, and eradication measures. Unmet need for health services is defined as the difference between health services considered necessary for a specific health problem and services actually received (2). Research indicates that unmet health needs can be influenced by immutable factors such as gender, age, ethnicity, and factors that can be influenced, including education, social factors, and predictors of healthcare accessibility (3). Unmet health needs are crucial for analyzing the health potential of the population. Analyzing the unmet health needs of the older adult is key to evaluating barriers to the implementation of services in the health and social care sector (4). Demographic changes have led to an increase in the older adult population globally, with individuals of older age at greater risk of inequality in healthcare system accessibility (5–7). Research results on unmet health needs in Europe suggest that material status and social support are important predictors influencing the realization of health needs (7–11). Older adult individuals often have multiple chronic diseases and comorbidities, which can significantly worsen due to unmet health needs (8–11). Health potential is lower in individuals between 65 and 80 years, where unmet health needs are present (12). The aim of this research study is to assess the relationship between demographic and socioeconomic predictors and the unmet health needs of the older adult population in Serbia. Unmet needs for health services are recognized as the most important indicator of the accessibility of health care for the older adult, they are influenced by various predictors such as age, place of residence, material status and social factors.

This research is important for the planning of health policies and the organization of the health system, which will prevent health inequalities in this population.

## 2 Methodology

### 2.1 Type of study

The research study is part of the fourth National Population Survey of the health status of the citizens of the Republic of Serbia, which was conducted by the Republic Institute of Statistics, Institute for Public Health of Serbia “Dr. Milan Jovanović Batut” and the Ministry of Health of the Republic of Serbia. The research was designed according to the methodology and instruments of the European Health Interview (13, 14).

### 2.2 Target population

With this research, the population of the older adult population of the Republic of Serbia was observed. All respondents are 65 years of age and older. The study does not include persons living in the territory of Kosovo and Metohija, as well as persons housed in collective households and specialized institutions (persons serving prison sentences, penal institutions, psychiatric institutions, institutions for the care and accommodation of the older adult, and monasteries). A stratified two-stage sample of the population of the Republic of Serbia was selected to show the representative health characteristics of the population at the level of four geographical regions (Belgrade, Vojvodina, Šumadija and Western Serbia, Southern and Eastern Serbia). These regions represent the main layers in the sample. Stratification was performed according to the place of residence of the residents; they were divided into respondents from rural and urban areas.

### 2.3 Characteristics of the sample

The sample size was calculated based on the requirements and in accordance with the recommendations of EUROSTAT for conducting health surveys of the population of Serbia (13, 14). Stratification was carried out according to the type of settlement (urban and other) and in four regions: Belgrade Region, Vojvodina Region, Šumadija and Western Serbia Region, and South and Eastern Serbia Region. The sample selection framework is based on the official population census of the Republic of Serbia from 2011. Census circles were used as the basis for sampling, which were formed specifically for the purposes of conducting the census. Representatives of each stratum participated in those census circles, defined as primary sample units. Selection was done systematically, with the probability of selection proportional to the size of each stratum. The measure of size was the number of households in each enumeration circle. The analysis of the results of this research is based on a sample of 3,540 inhabitants in 2019.

### 2.4 Time period of data collection

The research was carried out over 3 months, during October, November, and December in 2019, in accordance with the recommendations and rules of the European Population Health Survey.

### 2.5 Ethical and legal aspects

The ethical and legal aspects of the research were carefully managed to ensure compliance with legal and ethical norms. In health research, the international Declaration of Helsinki complied with ethical standards and there were no deviations from the principles of scientific research work. The research participants were provided with a written document that contained all the necessary information about the purpose of the study, the scope of their rights and the telephone number intended for additional information or possible complaints. All participants were informed about everything through a written notification. The respondents voluntarily gave their consent to participate in the research, by signing the informed consent. Anonymity of the participants during the research was ensured by not using data that could identify an individual, in accordance with the Law. This approach ensures compliance with ethical standards and rules, thus guaranteeing the impossibility of data misuse. The research results are published in aggregate form, which fully ensures the confidentiality of individual data. Consent was obtained for the use of data for scientific research purposes and publication of data in scientific journals by the Ethics Committee of the Institute for Public Health of Serbia “Dr. Milan Jovanović Batut” (Decision number: 5179/1).

### 2.6 Research instrument

The standardized questionnaire of the European Health Interview (EHIS) was used as a research instrument. The focus of our research were the questions from the questionnaire related to unmet needs for health care, the reasons for the emergence of unmet health needs and their connection with the socioeconomic information obtained from this questionnaire. Unmet health care needs were observed through the following variables from the questionnaire, whether in the last 12 months the necessary health care was missing or there was no need for health care services, reasons for unmet health care needs, due to a long wait for a scheduled medical examination, distance and problems with transportation, financial reasons. The financial status of respondents was analyzed in relation to household income, the respondents were classified from the lowest to the highest value of household income into five categories (quintiles), so that the first quintile contains the poorest and the fifth the wealthiest residents (13, 14).

### 2.7 Work in the field and monitoring the success of data collection

The interviewers were provided with appropriate training during which they received work instructions. There were 70 teams, each team covering an appropriate field area. Each team consisted of two members, one health worker, nurse or doctor, and one interviewer, with experience in conducting survey research. Sixteen field supervisors were in charge of supervising and controlling work in the field. On average, each supervisor hired to conduct the research supervised four teams. Monitoring of the research was continuous and included control of sampling and control of work in the field. The Republic Institute of Statistics conducted work control by direct contact with households on 25% of the total sample: 15% of households were contacted by phone, while 10% were contacted through field control, by visiting the household. The results of the conducted monitoring came to the conclusion that the entire course of realization of data collection was realized according to the given guidelines and in an appropriate manner (13, 14).

### 2.8 Statistical methods

The statistical program package SPSS 23.0 (Statistical Package for the Social Sciences) on the Windows platform was used in data processing. Descriptive and inferential statistical methods were used in data description/analysis. The data were of a categorical type, so the structure indicators, expressed as percentages, were used to describe the data. Applied methods of inferential statistics were χ^2^ test and logistic regression. In the analysis of the distribution of one characteristic, the χ^2^ test was used in the form of the agreement test. Testing the difference in the distribution of two or more observation features was performed with the χ^2^ test in the form of rxk-type contingency tables. The relationship between the dependent variable and a series of independent variables was tested by bivariate and multivariate logistic regression. First, all variables were tested in the univariate model, and then only the statistically significant variables (*p* ≤ 0.05) in the univariate regression were included in the multivariate model. The risk was assessed using the OR (odds ratio) size with a 95% confidence interval. The results are presented tabularly. All results with a probability of up to 5% (*p* ≤ 0.05) were considered statistically significant.

## 3 Results

3,540 respondents participated in the research, 1,528 (43.2%) male and 2,012 (56.8%) female (χ^2^ = 66.17, df = 1, *r* < 0.01). The average age of the respondents was 73.9 ± 6.3 years. The representation of individual age categories is significantly different (χ^2^ = 19.99, df = 1, *r* < 0.01). The largest number of respondents, 29.9%, is aged 65–69, and the smallest is 80 and over, 19.5%. The representation of respondents aged 70–74 and 75–79 is the same, 25.3%. Slightly more than half of the respondents live in a married union. Respondents live more often in urban areas, 53.8% (χ^2^ = 19.99, df = 1, *r* < 0.01), most often in the region of Šumadija and Western Serbia, 28.6%, and least often in Belgrade, 20.4% (χ^2^ = 49.19, df = 3, *r* < 0.01). More than half of the respondents have no more than primary school education. Every third respondent has completed secondary school, and only every seventh respondent has a higher education (χ^2^ = 882.71, df = 2, *r* < 0.01). 55.8% are poor, and 26.4% are rich (χ^2^ = 842.32, df = 2, *r* < 0.01). In the previous 12 months, 33.6% of women and 28.8% of men did not receive the necessary health services (χ^2^ = 29.03, df = 1, *r* < 0.01). Variations in the frequency of unmet health needs are present in different age groups (χ^2^ = 17.04, df = 6, *r* < 0.05). For respondents aged 65–69, 15.7% of them had no need for health care in the previous year. Among the oldest, 28.7% did not realize the necessary health services. The representation of unfulfilled health needs is similar among respondents who live in a married/cohabiting union and among respondents who live without a partner, divorced, widowed, or never married/married (χ^2^ = 1.2, df = 6, *r* > 0.05). The frequency of unmet health needs is almost identical in urban and other areas, but not among regions (χ^2^ = 99.75, df = 6, *r* < 0.01). In Šumadija and Western Serbia, 22.7% of respondents did not receive the necessary form of health care in the previous 12 months, in Southern and Eastern Serbia 28.7%, in Vojvodina 36.8%, in Belgrade the highest, 40.9% (Table 1).

**Table 1:** Unmet health needs in relation to the socioeconomic and demographic characteristics of the respondents.

Characteristics	Unmet health needs		No health care was needed	*р*
	Yes	No		
	(*n*, %)	(*n*, %)	(*n*, %)	
Gender				
Male	440 (28,8)	840 (55)	248 (16,2)	<0.01
Female	676 (33,6)	1,236 (56)	210 (10,4)	
Age categories				
65–69	344 (32,5)	548 (51,8)	166 (15,7)	<0.05
70–74	284 (31,7)	506 (56,4)	107 (11,9)	
75–79	290 (32,4)	509 (56,9)	95 (10,6)	
80 and over	198 (28,7)	403 (58,3)	90 (13)	
Marital status				
Married/cohabiting	611 (31,1)	1,086 (55,3)	268 (13,6)	>0.05
Singles	505 (32,1)	880 (55,9)	190 (12,1)	
Type of settlement				
Urban	600 (31,5)	1,080 (56,8)	223 (11,7)	>0.05
The others	516 (31,5)	886 (54,1)	235 (14,4)	
Region				
Vojvodina	326 (36,8)	425 (48)	135 (15,2)	<0.01
Belgrade	296 (40,9)	352 (48,7)	75 (10,4)	
Šumadija and Western Serbia	230 (22,7)	665 (65,7)	117 (11,6)	
Southern and Eastern Serbia	264 (28,7)	524 (57)	131 (14,3)	
Degree of education				
No School/primary school	669 (34,2)	1,059 (54,1)	228 (11,7)	<0.05
High School	307 (29,1)	598 (56,7)	150 (14,2)	
College/University	140 (26,5)	309 (58,4)	80 (15,1)	
Material condition				
Poor	674 (34,1)	1,042 (52,8)	259 (13,1)	<0.05
Average	184 (29,2)	367 (58,2)	80 (12,7)	
Rich	258 (27,6)	557 (59,6)	119 (12,7)	

χ^2^ test.

34.2% of the respondents received the necessary health care in the previous year among the respondents who did not go to school or had completed elementary school. Furthermore, as the level of education increases, so does the percentage of respondents who did not receive the necessary form of health care, 29.1% of them with secondary education and 26.5% with higher education (χ^2^ = 18.21, df = 4, *r* < 0.05). Unmet medical needs in the previous 12 months were experienced by 34.1% of the poor, i.e., 29.2% of respondents with an average financial condition and 27.69% of the rich (χ^2^ = 16.33, df = 4, *r* < 0.05) (Table 1).

Univariate analysis identified as predictors of unmet health needs, gender (OR = 1.18; 95% CI = 1.02–1.37), region (OR = 1.24; 95%, CI = 1.16–1.32), level of education (OR = 0.83; 95%, CI = 0.75–0.81), and material condition (OR = 0.84; 95% CI = 0.77–0.92). Multivariate regression analysis of demographic characteristics that showed statistical significance in the univariate model overall explained between 4.2% (Cock & Snell R Square) and 5.9% (Nagelkerke R Square) of the variance in unmet health needs and correctly classified 66.3% of cases. Statistically significant demographic predictors were the region in which the respondents live, the level of education and material condition. The strongest predictors are the region where the respondents live and their financial situation. Respondents from Vojvodina report unfulfilled health needs 1.7 times, and those from Belgrade 2.3 times more often than residents of southern and eastern Serbia. However, the frequency of unaccomplished health care among residents of Šumadija and Western Serbia is 0.7 times lower than among residents of southern and eastern Serbia. Respondents with the lowest level of education, primary school, do not receive the necessary health services 1.5 times more often than highly educated respondents. Respondents who, according to the well-being index, fall into the category of poor, have 1.6 times higher chances of not receiving the necessary health services compared to the rich. There is a similar difference in the realization of the necessary health care among the rich and respondents of average financial condition (Table 2).

**Table 2:** Univariate and multivariate model analysis of the association of sociodemographic predictors with the occurrence of unmet health needs.

Categories (referential)	Univariate model				Multivariate model			
	B	SE	ОR (95% CI)	*р*	B	SE	ОR (95% CI)	*р*
Gender (female)								
Male	−0.77	0.06	1.18 (1.02–1.37)	<0.05	−0.05	0.08	>0.05	
Age range (80 and more)					**/**			
65–69	0.19	0.11	1.21 (0.98–1.5)	>0.05				
70–74	0.11	0.11	1.12 (0,9-1,4)					
75–79	0.13	0.11	1.14 (0.91–1.41)					
Marital status (married)					/			
Singles	0.03	0.07	1.03 (0.89–1.19)	>0.05				
Type of settlement (urban)					/			
The others	0.02	0.07	1.02 (0.88–1.18)	>0.05				
Region (Southern/Eastern Serbia)								
Vojvodina	0.43	0.1	1.54 (1.26–1.89)	<0.01	0.48	0.1	1.67 (1.32–1.98)	<0.01
Belgrade	0.55	0.11	1.74 (1.41–2.14)		0.848	0.12	2.33 (1.85–2.92)	<0.01
Sumadija/Western Serbia	−0.32	0.11	0.73 (0.59–0.89)		−0.38	0.11	0.74 (0.6–0.92)	<0.05
Education (College/University)								
Primary school	0.36	0.11	1.44 (1.16–1.79)	<0.05	0.38	0.13	1.46 (1.13–1.88)	<0.05
High School	0.14	0.12	1.15 (0.9–1.46)		0.12	0.13	1.13 (0.88–1.44)	>0.05
Material condition (rich)								
Poor	0.33	0.09	1.39 (1.17–1.65)	<0.01	0.46	0.11	1.58 (1.23–1.94)	<0.01
Average	0.08	0.12	1.08 (0.86–1.35)		0.2	0.12	1.22 (0.96–1.55)	>0.05

B, Beta coefficient; SE, Standard error; OR, Odds Ratio; and CI, Confidence interval.

In the Republic of Serbia, the largest number of residents aged 65 and over achieved the necessary form of health care, according to the results of the latest National Survey of the Health of the Residents of the Republic of Serbia. 31.5% of respondents gave an affirmative answer to the question of whether in the previous 12 months they did not receive the necessary health care on time, due to a long wait for an appointment, distance/problems with transportation or financial difficulties. Slightly more than half of the respondents, 55.5%, received all the necessary health care, while 12.9% had no need for health care in the previous 12 months. The distribution of the reasons for missing the necessary health care, waiting lists, distance from the health care facility or financial constraints, is different (χ^2^ = 7.03, df = 2, *r* < 0.05). Every fourth respondent, 23.2%, cites financial difficulties as the reason for the lack of necessary health services. Every sixth respondent in the previous year did not receive the necessary health care on time due to a long wait for an appointment, 15.8%. 8.3% of the respondents cited problems with transportation/distance to the health care facility as the reason for the unmet need for health care (Table 3).

**Table 3:** Causes of unmet need for health care.

Reasons for absence	*n*	%	*р*
A long wait for a scheduled medical examination	558	15,8	<0.05
Distance/transportation problems	294	8,3	
Financial reasons	820	23,2	

χ^2^ test.

Further analysis shows that out of a total of 705 respondents who cited one reason for missing the necessary health care, 212 (30.1%) of them cited waiting lists, 39 (5.5%) of them distance, and 455 (64.4%) of them finances.

All three reasons were mentioned by 146 respondents. Furthermore, out of a total of 264 respondents who gave two reasons for the lack of necessary health services, 42 (15.9%) of them did not receive the necessary health care due to the waiting list and distance, 63 (23.9%) due to distance and financial limitations, and 159 (60.2%) due to the waiting list and finances.

## 4 Discussion

Unmet health needs are an important sign of inequality in access to health care. The presence of unfulfilled health needs of the older adult population is a major public health problem for this population. The older adult population has numerous chronic diseases and comorbidities, if the presence of unfulfilled health needs is high, it will lead to a drop in the level of health potential and worsening of chronic diseases, which puts this population at risk for health complications or death. Socio-economic predictors that influence the emergence of inequality in the availability of health care should be of a low level and be prevented by proper organization of the health system and planning of health policies (15). The older adult population often has more complex and frequent needs for health care compared to younger people, certain researchers come to the result that the unfulfilled health needs of the older adult population increase the risk of death within 5 years with an odds ratio of 1.64. Regardless of this worrisome data, research into the unfulfilled health needs of the older adult population is not so common in practice (16). Across the countries of Europe, there are different data on unfulfilled health needs, the prevalence of health care needs is different and depends on numerous social factors and the organization of the health system. In any case, unmet health needs are based on numerous predictors that negatively affect people’s health (17). In our study, a large number of respondents from the population of the older adult population did not meet their health needs (31%), this data can also be found in more developed countries of Europe, such as France, whose research on the unfulfilled health needs of the older adult population leads to the result that this a problem present in as much as one-quarter of the population of the older adult population. An identical predictor of unmet health needs is the socioeconomic position of the older adult, which puts them at risk for inequalities in the availability of health care (16). The data obtained and presented in our research are similar to the data from the Health Survey of the Republic of Croatia. In this country, which culturally and according to the organization of health care is very similar to the Republic of Serbia, as many as 26.3% of the population were in a situation where they had unfulfilled health needs. The largest share of the population in the observed sample are those who could not, for financial reasons, obtain a particular form of health care. The dominant unfulfilled health needs of the inhabitants of the Republic of Croatia are in the field of dental health care (11.1%) (18). The results of our research show results related to numerous predictors related to the community, the position of the older adult, the availability of health care, and the impact of health policy, but also related to numerous predictors related to the strength of the individual and his personal characteristics, social economic differences and lifestyle, which has been the subject of numerous other scientific studies (16). Research on the unmet health needs of the older adult population in Germany also comes to the conclusion that this problem is common in the population of older citizens. Unmet health needs are often related to mental function, physical health, and mobility. Compared to earlier studies in Germany in 2019, unmet health needs tend to decrease (6). This data differs from our results, in the Republic of Serbia in 2013, 26.4% of respondents did not receive the necessary form of health care in the previous 12 months, and in 2019, 31.5%. Researchers who analyzed the unmet health needs of the older adult population in Korea and China also came to the conclusion that social support and lifelong health promotion can prevent the consequences of unmet health needs of this population (19–21). The predictor of unfulfilled health needs of the older adult population, which is also dominant in our research, is financial barriers for aging health care needs, this data are identical in numerous other analyses of the European population. We reach similar results in the aspect of the unfulfilled health needs of the older adult population due to the distance and inaccessibility of health care due to the impossibility of transportation to a health facility and similar reasons. In a large number of cases, unmet health needs are influenced by the place of residence, rural or urban environment, the largest number of health institutions are concentrated in the urban environment (22). The results of our research are also similar to the results obtained in the national survey of unmet health needs in Greece, where one of the most dominant predictors of the emergence of unmet health needs is of a social-economic nature, that is, the existence of financial reasons for unmet needs for health care. In the research of unfulfilled needs in Greece, emphasis is also placed and recommendations are made for a more careful creation of health policy that would lead to a reduction of inequality in the accessibility and availability of health care for all citizens (23). Numerous other researches also come to similar results, where financial reasons and the inaccessibility of health care are the main reasons for the emergence of unfulfilled health needs of the older adult population. Older adult people have lower incomes and are often not able to travel independently and move to a health facility (24–26). Unmet health needs, regardless of the predictor that caused them, can significantly negatively affect the health potential of an older adult person and lead to numerous complications of the health condition in certain cases and to complications associated with the death of an older adult person (12). Unfulfilled needs for health care are key indicators for monitoring the degree of inequality in access, use, and realization of health care (27).

## 5 Conclusion

The unmet health needs of the older adult population have a negative impact on the health potential of this population, as well as on their equality in access to health care. One in three oldest residents did not receive the necessary health care in the previous year, most often due to financial limitations (one in five) or a waiting list (one in six). The lack of finances most often causes the absence of medical health care, but also the procurement of medicines. Compared to the waiting lists and the distance to the health care facility, finances are the main cause of the increase in the non-availability of health care.

## Data availability statement

The raw data supporting the conclusions of this article will be made available by the authors, without undue reservation.

## Ethics statement

The studies involving humans were approved by Institute for Public Health of Serbia “Dr. Milan Jovanovic Batut” Belgrade, Serbia, Ethics Committee of the Institute, Number of consent 5179/1. The studies were conducted in accordance with the local legislation and institutional requirements. Written informed consent for participation was not required from the participants or the participants’ legal guardians/next of kin in accordance with the national legislation and institutional requirements.

## Author contributions

NS: Conceptualization, Data curation, Formal analysis, Funding acquisition, Investigation, Methodology, Project administration, Resources, Software, Supervision, Validation, Visualization, Writing – original draft, Writing – review & editing. SR: Conceptualization, Data curation, Formal analysis, Methodology, Project administration, Supervision, Validation, Writing – review & editing. VJ: Methodology, Supervision, Validation, Writing – review & editing, Data curation, Formal analysis. NR: Data curation, Formal analysis, Methodology, Visualization, Writing – review & editing. IL: Formal analysis, Methodology, Writing – review & editing. SB: Supervision, Writing – review & editing. BA: Methodology, Supervision, Writing – review & editing. BiB: Data curation, Formal analysis, Investigation, Writing – review & editing. AM: Methodology, Supervision, Writing – original draft. AA: Data curation, Methodology, Visualization, Writing – original draft. BoB: Supervision, Writing – review & editing. SV: Data curation, Methodology, Supervision, Writing – original draft. SK: Supervision, Writing – original draft, Writing – review & editing.
